# Editorial: Minding Glial Cells in the Novel Understandings of Mental Illness

**DOI:** 10.3389/fncel.2017.00048

**Published:** 2017-02-28

**Authors:** Takahiro A. Kato, Aye M. Myint, Johann Steiner

**Affiliations:** ^1^Department of Neuropsychiatry, Graduate School of Medical Sciences, Kyushu UniversityFukuoka, Japan; ^2^Brain Research Unit, Innovation Center for Medical Redox Navigation, Kyushu UniversityFukuoka, Japan; ^3^Department of Psychiatry, Ludwig-Maximilians-UniversityMunich, Germany; ^4^Department of Psychiatry, Otto-von-Guericke University MagdeburgMagdeburg, Germany

**Keywords:** microglia, psychiatric disorders, depression, schizophrenia, autism, astrocytes, oligodendrocytes, autism spectrum disorders

During the last few decades, it has been assumed that dysfunctions of neurons and neuronal networks including synaptic abnormalities and consecutive disturbances of neurotransmitters are the main and sole causes of psychiatric disorders. Recent neuroscience has revealed various previously unknown roles of glial cells such as astrocytes, oligodendrocytes, and microglia as modulators of neurotransmission. These glial cells have proved to continuously contact with neurons/synapses, and have been shown to play important roles in brain development, homeostasis, and various brain functions. Beyond the classic neuronal doctrine of neuropsychiatry, accumulating evidence has suggested that abnormalities and disturbances of the crosstalk between neurons and glial cells may induce mental dysfunction and be a risk factor for the manifestation of psychiatric disorders. However, these mechanisms have yet to be well-understood.

This research topic of “the Frontiers in Cellular Neuroscience” has focused on the most recent developments and ideas in the study of glial cells (astrocytes, oligodendrocytes, and microglia) targeting psychiatric disorders such as schizophrenia, mood disorders, and autism. Here, we publish more than 20 articles including original research, review, perspective, and commentary. While all of the articles are focused on psychiatric disorders, a variety of methods/approaches have been employed from molecular, cellular, and pharmacological approaches using *in vitro*/*in vivo* experimental methods to translational approaches using human tissues.

Human postmortem research using brain tissues of patients with psychiatric disorders is one of the most important research approaches in biological psychiatry. In this research topic, Bernstein et al. reported a reduced density of glutamine synthetase immunoreactive astrocytes in different cortical areas in major depression but not in bipolar I disorder. Falkai et al. revealed an interaction between a decrease of oligodendrocyte and interneuron density in the hippocampus of schizophrenia patients and discussed the association of oligodendrocyte number with cognitive deficits, proposing that a decreased number of oligodendrocytes in the anterior, and entire hippocampus may be involved in cognitive deficits by impairing the connectivity of this structure in schizophrenia.

On the other hand, various novel approaches of brain imaging techniques have been developed to reveal glial dysfunctions in living patients with psychiatric disorders (Kato et al., 2013a). Peripheral blood markers are also suggested to be useful biomarkers of psychiatric disorders. For example, S100B has been considered as a glial marker protein, particular to oligodendrocytes and astrocytes. It passes the blood brain barrier and is detectable in peripheral blood. In this research topic, Schumberg et al. reported that serum S100B is related to illness duration and clinical symptoms in schizophrenia by a meta-regression analysis. Furthermore, combination analysis between brain imaging data and blood data is becoming one of the most highlighted approaches in psychiatry. Milleit et al. conducted an association analysis between brain imaging data of voxel based morphometry (VBM) and serum S100B concentrations in unmedicated patients with schizophrenia and healthy volunteers, and revealed that serum S100B protein is specifically related to white matter changes in patients with schizophrenia. This report suggests the involvement of S100B in an ongoing and dynamic process associated with structural brain changes and brain connectivity in schizophrenia.

As mentioned above, peripheral blood markers including components/materials of serum, plasma, and genes are possible useful biomarkers of psychiatric disorders. In addition, blood cells themselves have also been highlighted as possible biomarkers. Microglia and peripheral monocytes (monocytic cells) are both of mesodermal origin. Takahashi et al. discussed this linkage focusing on the activation of microglia and peripheral monocytes to understand the pathophysiology of psychiatric disorders. Ohgidani et al. introduced a novel translational tool for neuropsychiatric disorders, called “induced microglia-like (iMG) cells,” which can be produced within 2 weeks from fresh human monocytes by adding only two cytokines (GM-CSF and IL-34). They have recently reported on the suitability of the iMG cells to study and understand microglial pathophysiology in patients with schizophrenia and bipolar disorder (Sato-Kasai et al., 2016; Ohgidani et al., 2017). Alternatively, human induced pluripotent stem (iPS) cells- and embryonic stem (ES)-oriented microglia-like cells (termed pMGLs) may also prove to be suitable tools (Muffat et al., 2016). We believe that both the generation of iMG cells and pMGLs will provide a strong method to reveal the potential contribution of microglial cells in psychiatric disorders in more detail.

In spite of great advances in human research tools as shown above, animal models are still thought to be essential for psychiatric research. Epidemiological studies suggest that prenatal exposure to bacterial and viral infection is an important environmental risk factor for schizophrenia. The maternal immune activation (MIA) animal model is used to study how an insult directed at the maternal host can have adverse effects on the fetus, leading to behavioral and neurochemical changes later in life. In this research topic, de Souza et al. observed an upregulation of astroglial markers (S100B and GFAP) in a MIA model of schizophrenia by LPS in Wistar rats; the brain-regional expression pattern was sex-dependent. Smolders et al. reported that MIA evoked by polyinosinic:polycytidylic acid (polyI:C) does not evoke microglial cell activation in the embryo. This report suggests that the behavioral and neurological alterations in offspring cannot be related to the alteration of the activation state of embryonic microglial cells. Their *in vitro* studies also indicated that microglia cannot be directly activated by poly (I:C) or IL-6 exposure. However, recent studies in other research groups indicate that there is an increase in microglial density in different brain regions in the adult poly (I:C) MIA offspring (postnatal and adult age) (Juckel et al., 2011; Manitz et al., 2013). As commentary responding to Smolders et al., Bernstein et al. suggested the role of astrocytic activation in the brains of MIA offspring. Further investigations are needed to understand the relevance of microglial and astrocytic activation in each developmental stage of the MIA models.

On the other hand, the Cuprizone-treatment rodent model, one of the classical models of multiple sclerosis (MS), is now regarded as a useful model of schizophrenia (Xiao et al., 2008), showing a series of dysfunctions of glial cells such as microglia, astrocytes, and oligodendrocytes. Wang et al. reported that quetiapine inhibits microglial activation by neutralizing abnormal STIM1-mediated intercellular calcium homeostasis and promoting myelin repair in a cuprizone-induced mouse model of demyelination. Interestingly, Gentile et al. proposed the merit of utilizing the behavioral and neuro-glial studies with experimental autoimmune encephalomyelitis (EAE)—the most famous animal model of MS in order to explore the role of microglia in mood disorders.

This special issue also contains two mice model studies focusing on anxiety and glial cells. Zimmer et al. demonstrated that long-term administration of memantine (a NMDAR antagonist) induced anxiety-like behaviors, and decreased glutamate uptake activity in both the frontoparietal cortex and hippocampus without altering the immunocontents of the astroglial glutamate transporters GLT-1 and GLAST. Chen et al. reported that impairment of oligodendroglia maturation leads to aberrantly increased cortical glutamate and anxiety-like behaviors in juvenile mice.

High throughput technology of genome analysis, especially genome-wide association study (GWAS), has shown various candidate genes of psychiatric disorders such as schizophrenia and bipolar disorder (Psychiatric GWAS Consortium Bipolar Disorder Working Group, 2011; Ripke et al., 2013; Schizophrenia Working Group of the Psychiatric Genomics, 2014). Apart from genome analyses, proteome and metabolome analyses are expected to reveal unknown biological aspects of psychiatric disorders (Kaddurah-Daouk and Krishnan, 2009; Domenici et al., 2010; Setoyama et al., 2016). In this research topic, Davalieva et al. reviewed the recent advances of proteomic research in schizophrenia. Three original research articles applying the proteomic analysis of glial cells with a pharmacological intervention are also included in this topic. Guest et al. reported that MK-801 treatment affects glycolysis in oligodendrocytes more than in astrocytes and neuronal cells. Cassoli et al. compared effects of MK-801 and the antipsychotic drug clozapine on the proteome of cultured oligodendrocytes. In addition, focusing in a hypothesis-driven study on energy metabolism, Steiner et al. compared the typical antipsychotic drug haloperidol with the atypical antipsychotic compound clozapine; only the latter promoted glycolysis and myelin lipid synthesis in cultured oligodendrocytes. These data suggest that psychotropic drugs, originally developed for the modulation of neurons and/or synaptic neurotransmission, are also acting on astrocytes and oligodendrocytes. Similarly, recent *in vitro* studies using rodent microglial cells have revealed that psychotropic drugs, especially antipsychotics and antidepressants may also directly modulate microglial cells (Kato et al., 2011, 2013b), however the underlying signal transduction mechanisms have not been well clarified. In this research topic, Mizoguchi et al. proposed that microglial intracellular Ca^2+^ signaling may be an important target of antipsychotic actions. On the other hand, epigenetic mechanisms may be important disease modifiers. In line with this idea, Chen et al. introduced the recent topic of DNA methylation in glial cells for further research of schizophrenia.

In this research topic, we have included several review papers proposing interesting theories and hypotheses linking psychiatric disorders with dysfunctions of glial cells: Yamamuro et al. introduced potential primary roles of glial cells in the underlying mechanisms of psychiatric disorders. Petrelli et al. discussed the possible link between autism spectrum disorders and glial cells especially astrocytes and microglia. Koyama introduced functional alterations of astrocytes in mental disorders, and proposed pharmacological modulation of astrocytes as a novel drug target. Noda depicted a possible role of glial cells in the relationship between thyroid dysfunction and mental disorders. And finally, Stokes et al. discussed the recent evidence for an involvement of microglial and/or astrocytic P2X7 in the pathophysiology of depressive disorders.

We are very pleased to publish a broad spectrum of papers focusing on glial cells in the field of psychiatry utilizing multi-dimensional approaches. We believe that further investigations to clarify the correlation between glial cells and psychiatric disorders will contribute to a novel understanding of the pathophysiology of mental illnesses and the development of effective treatment strategies.

## Author contributions

All authors listed, have made substantial, direct and intellectual contribution to the work, and approved it for publication.

### Conflict of interest statement

The authors declare that the research was conducted in the absence of any commercial or financial relationships that could be construed as a potential conflict of interest.

## References

[B1] Domenici E. Wille D. R. Tozzi F. Prokopenko I. Miller S. Mckeown A.. (2010). Plasma protein biomarkers for depression and schizophrenia by multi analyte profiling of case-control collections. PLoS ONE 5:e9166. 10.1371/journal.pone.0009166 20161799 PMC2820097

[B2] Juckel G. Manitz M. P. Brune M. Friebe A. Heneka M. T. Wolf R. J. (2011). Microglial activation in a neuroinflammational animal model of schizophrenia – a pilot study. Schizophr. Res. 131, 96–100. 10.1016/j.schres.2011.06.018 21752601

[B3] Kaddurah-Daouk R. Krishnan K. R. (2009). Metabolomics: a global biochemical approach to the study of central nervous system diseases. Neuropsychopharmacology 34, 173–186. 10.1038/npp.2008.174 18843269

[B4] Kato T. A. Monji A. Mizoguchi Y. Hashioka S. Horikawa H. Seki Y. (2011). Anti-Inflammatory properties of antipsychotics via microglia modulations: are antipsychotics a “fire extinguisher” in the brain of schizophrenia? Mini Rev. Med. Chem. 11, 565–574. 10.2174/138955711795906941 21699487

[B5] Kato T. A. Watabe M. Kanba S. (2013a). Neuron-glia interaction as a possible glue to translate the mind-brain gap: a novel multi-dimensional approach toward psychology and psychiatry. Front. Psychiatry 4:139. 10.3389/fpsyt.2013.00139 24155727 PMC3804762

[B6] Kato T. A. Yamauchi Y. Horikawa H. Monji A. Mizoguchi Y. Seki Y.. (2013b). Neurotransmitters, psychotropic drugs and microglia: clinical implications for psychiatry. Curr. Med. Chem. 20, 331–344. 10.2174/0929867311320030003 23157624

[B7] Manitz M. P. Esslinger M. Wachholz S. Plumper J. Friebe A. Juckel G.. (2013). The role of microglia during life span in neuropsychiatric disease–an animal study. Schizophr. Res. 143, 221–222. 10.1016/j.schres.2012.10.028 23182726

[B8] Muffat J. Li Y. Yuan B. Mitalipova M. Omer A. Corcoran S.. (2016). Efficient derivation of microglia-like cells from human pluripotent stem cells. Nat. Med. 22, 1358–1367. 10.1038/nm.4189 27668937 PMC5101156

[B9] Ohgidani M. Kato T. A. Haraguchi Y. Matsushima T. Mizoguchi Y. Murakawa-Hirachi T.. (2017). Microglial CD206 gene has potential as a state marker of bipolar disorder. Front. Immunol. 7:676. 10.3389/fimmu.2016.00676 28119691 PMC5220016

[B10] Psychiatric GWAS Consortium Bipolar Disorder Working Group (2011). Large-scale genome-wide association analysis of bipolar disorder identifies a new susceptibility locus near ODZ4. Nat. Genet. 43, 977–983. 10.1038/ng.943 21926972 PMC3637176

[B11] Ripke S. O'dushlaine C. Chambert K. Moran J. L. Kahler A. K. Akterin S.. (2013). Genome-wide association analysis identifies 13 new risk loci for schizophrenia. Nat. Genet. 45, 1150–1159. 10.1038/ng.2742 23974872 PMC3827979

[B12] Sato-Kasai M. Kato T. A. Ohgidani M. Mizoguchi Y. Sagata N. Inamine S.. (2016). Aripiprazole inhibits polyI:C-induced microglial activation possibly via TRPM7. Schizophr. Res. 178, 35–43. 10.1016/j.schres.2016.08.022 27614570

[B13] Schizophrenia Working Group of the Psychiatric Genomics C. (2014). Biological insights from 108 schizophrenia-associated genetic loci. Nature 511, 421–427. 10.1038/nature13595 25056061 PMC4112379

[B14] Setoyama D. Kato T. A. Hashimoto R. Kunugi H. Hattori K. Hayakawa K.. (2016). Plasma metabolites predict severity of depression and suicidal ideation in psychiatric patients-a multicenter pilot analysis. PLoS ONE 11:e0165267. 10.1371/journal.pone.0165267 27984586 PMC5161310

[B15] Xiao L. Xu H. Zhang Y. Wei Z. He J. Jiang W.. (2008). Quetiapine facilitates oligodendrocyte development and prevents mice from myelin breakdown and behavioral changes. Mol. Psychiatry 13, 697–708. 10.1038/sj.mp.4002064 17684494

